# Endoscopic Grading of Gastric Intestinal Metaplasia Using Magnifying and Nonmagnifying Narrow-Band Imaging Endoscopy

**DOI:** 10.3390/diagnostics12123012

**Published:** 2022-12-01

**Authors:** Masashi Kawamura, Tomoyuki Koike, Yohei Ogata, Ryotaro Matsumoto, Kota Yano, Takashi Hiratsuka, Hideaki Ohyama, Isao Sato, Kimiko Kayada, Suguo Suzuki, Satsuki Hiratsuka, Yumiko Watanabe

**Affiliations:** 1 Department of Gastroenterology, Sendai City Hospital, 1-1-1, Asutonagamachi, Taihaku-ku, Sendai 982-8502, Miyagi, Japan; 2 Division of Gastroenterology, Tohoku University Graduate School of Medicine, 1-1, Seiryo-machi, Sendai 980-8574, Miyagi, Japan

**Keywords:** gastric intestinal metaplasia, narrow-band imaging, image-enhanced endoscopy, magnifying endoscopy, endoscopic grading of gastric intestinal metaplasia, gastric cancer

## Abstract

Several endoscopic findings obtained by magnifying image-enhanced endoscopy (IEE) are reportedly correlated with gastric intestinal metaplasia (IM); however, the differences between magnifying and nonmagnifying IEE for the diagnosis of gastric IM remain unknown. This study included 100 consecutive patients who underwent narrow-band imaging endoscopy. Four areas of the stomach were evaluated using nonmagnifying and magnifying IEE. Light-blue crest (LBC), white opaque substance (WOS), and endoscopic grading of the gastric IM (EGGIM) were assessed. The concordance rates between nonmagnifying and magnifying IEE were 80.5% for LBC and 93.3% for WOS. The strength of agreement between each observation technique showed good reproducibility, with a kappa value of 0.69 and 0.83 for LBC and WOS, respectively. The individual EGGIM score indicated a good correlation between nonmagnifying and magnifying IEE (concordance rate, 75%; kappa value, 0.67). The prevalence of a high EGGIM score in patients with and without gastric cancer (GC) showed a significant difference both with nonmagnifying IEE (odds ratio (OR), 3.3; 95% confidence interval (CI), 1.2–9.0), and magnifying IEE (OR, 3.1; 95% CI, 1.1–8.9). Nonmagnifying IEE has the potential to stratify the individual risk of GC, similar to magnifying IEE, warranting further investigation with histological assessment.

## 1. Introduction

Intestinal metaplasia (IM) and the risk of gastric cancer (GC) are strongly associated [1]. However, because assessment of gastric IM is necessary for early diagnosis and treatment of GC, there exist various methods of assessing gastric IM. The gold-standard diagnostic method for gastric IM is histopathology. The operative link on gastric IM (OLGIM) classification [2] was proposed for the staging of gastritis and stratification of the risk of GC using endoscopic biopsy specimens. Patients with high OLGIM stages (III/IV) demonstrating an extensive intragastric distribution of histological IM are classified in the significantly high-risk group for GC [3,4].

Advanced image-enhanced endoscopy (IEE), including narrow-band imaging (NBI) endoscopy, is reportedly a more accurate diagnostic tool for gastric IM than conventional white-light endoscopy (WLE) [5]. In European countries, a newly proposed endoscopic classification for gastric IM assessment using IEE, namely, the endoscopic grading of gastric IM (EGGIM), is reportedly beneficial for GC risk stratification [6,7].

Several IEE findings have been shown to be strongly associated with gastric IM. Light-blue crest (LBC), a light-blue line observed on the epithelial surface during IEE, is considered a phenomenon associated with the epithelial brush border and is typical of gastric IM histopathology [8]. White opaque substance (WOS), a white mucosal epithelium observed during IEE, is reportedly associated with the accumulation of microscopic lipid droplets within the mucosa [9,10]. Tubulovillous microsurface structures and marginal turbid band (MTB) have also been reported to be associated with gastric IM [5,11,12,13].

These IEE findings that are strongly associated with gastric IM were originally reported using magnifying IEE; however, magnifying IEE cannot be used in all clinical settings. To the best of our knowledge, the clinical differences in the assessment of gastric IM between magnifying and nonmagnifying IEE, the latter of which is frequently used in general clinical practice, have yet to be clarified. This study was performed to clarify the differences in the assessment of gastric IM using magnifying and nonmagnifying IEE.

## 2. Materials and Methods

### 2.1. Study Populations

This observational study was performed in an endoscopy unit of a city hospital (Sendai City Hospital, Sendai, Miyagi, Japan). From August 2017 to March 2019, 100 consecutive patients who underwent gastroendoscopic examinations for any indication (e.g., abdominal symptoms, detailed examinations for abdominal findings) were enrolled in the study. Our exclusion criteria were bleeding, severe organ failure, and a history of stomach surgery. The *Helicobacter pylori* infection status was assessed via a combination of serum anti-*H. pylori* IgG antibody measurement, history of *H. pylori* eradication, and endoscopic findings using WLE. GC was confirmed by histological analysis of specimens. The study protocol was approved by the Institutional Review Board of Sendai City Hospital. The study protocol was explained to all the patients, and their written informed consent to participate in the study was obtained.

### 2.2. Endoscopic Procedure

**Figure 1:**
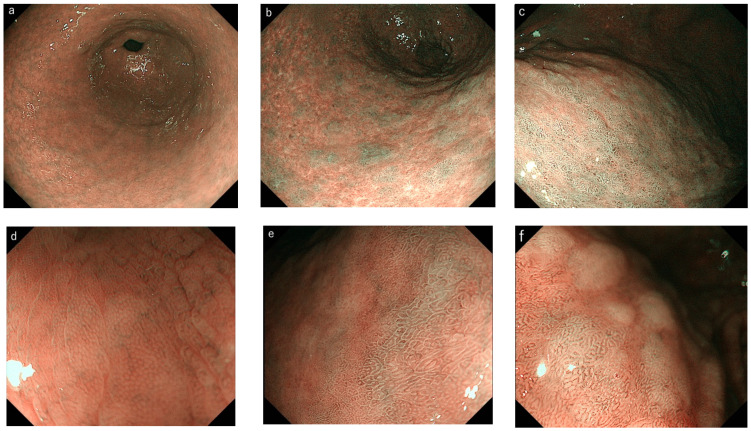
Examples of gastric intestinal metaplasia (IM) observations using (**a**–**c**) nonmagnifying and (**d**–**f**) magnifying image-enhanced endoscopy (IEE). (**a**) Score 0: normal gastric mucosa with nonmagnifying IEE. (**b**) Score 1: presence of focal gastric IM with nonmagnifying IEE (≤30% of the area). (**c**) Score 2: presence of extensive gastric IM with nonmagnifying IEE (>30% of the area). (**d**) Magnified view of the normal gastric mucosa. (**e**) Focal light-blue crest (LBC) and tubulovillous/marginal turbid band observed with magnifying IEE. (**f**) Extensive LBC and white opaque substance observed with magnifying IEE.

An endoscopist with experience performing more than 5000 upper endoscopy procedures (M.K.) performed the endoscopic examinations in this study using an EVIS LUCERA ELITE (CV-290) video system (OLYMPUS Co., Tokyo, Japan) and a high-resolution magnifying endoscope (Model H260Z or H290Z; OLYMPUS Co.) The structure-enhancement function of the video processor was set at B8 for NBI endoscopy, and the color mode was fixed at level 1. Following the application of topical anesthesia, the videoendoscope was inserted into the stomach, and routine observation using WLE was performed. Next, four areas (the lesser and greater curvatures of the antrum and corpus) were photographed using nonmagnifying NBI endoscopy. In addition, several images of the same areas were captured using NBI endoscopy with moderate to high magnification. The magnified areas were selected to represent the IEE images in the field (Figure 1). The incisura angularis was not photographed because of its questionable diagnostic benefit for assessing gastric IM [14].

### 2.3. Magnifying and Nonmagnifying IEE for Gastric IM

The captured images of magnifying and nonmagnifying IEE were evaluated by advanced-level endoscopists (>5000 upper endoscopies performed) (M.K. and Y.O.), intermediate-level endoscopists (2000–5000) (I.S. and S.S.), and trainee-level endoscopists (<2000) (S.H. and Y.W.). The images were randomly allocated to each physician and diagnosed for gastric IM (IEE-IM) on a computer screen. Before the start of the assessment, a lecture to agree upon a standard assessment for IEE-IM diagnosis was provided to all physicians. The extent of IEE-IM was classified into three categories according to the EGGIM [6]: none, focal (≤30% of the area), or extensive (>30% of the area).

First, we assessed the extent of LBC and WOS from the images of all the observed areas and investigated the concordance rate between magnifying and nonmagnifying IEE. Next, the extent of all the IEE-IM findings (LBC, WOS, or tubulovillous/MTB) in each of the four areas was scored as 0 (none), 1 (focal), or 2 (extensive) according to the EGGIM. The individual EGGIM scores were calculated from the sum of the IEE-IM scores. We evaluated the difference in the individual EGGIM scores between magnifying and nonmagnifying IEE. Finally, we investigated the prevalence of high EGGIM scores in patients with and without GC in magnifying and nonmagnifying IEE.

### 2.4. Statistical Analysis

Because this was an exploratory pilot observational study, the sample size was calculated based a previous study of diagnostic accuracy using nonmagnifying and magnifying endoscopy. Ezoe et al. [15] reported that the diagnostic accuracy for small gastric carcinomas was 64.8% with nonmagnifying endoscopy and 94.3% with magnifying IEE. Based on that study, our sample size was estimated to be 96 patients (48 patients in each group) with a margin of noninferiority set at 0.1, a one-sided α of 0.05, and a β of 0.20. Numerical data are shown as mean and standard deviation. The concordance rate and weighted kappa value were calculated to assess the differences in the prevalence of IEE-IM findings in magnifying and nonmagnifying observations. The prevalence of high EGGIM scores in patients with and without GC was compared using Pearson’s chi-squared test. A two-sided *p*-value of <0.05 was considered statistically significant. SPSS Statistics 17.0 (SPSS Inc., Chicago, IL, USA) was used for all data analyses.

## 3. Results

The patients’ baseline clinical characteristics are shown in Table 1. Twenty-one patients did not have *H. pylori* infection, 59 had current infection, and 20 had past infection. The endoscopic grading of atrophy (Kimura–Takemoto classification) [16] was none-to-mild (C0, C1) in 22 patients, moderate (C2, C3) in 46 patients, and severe (O1, O2, or O3) in 32 patients. GC was diagnosed in 21 patients via histopathology. Most patients with GC exhibited early-stage (pT1) disease and the differentiated histological type.

**Figure 2:**
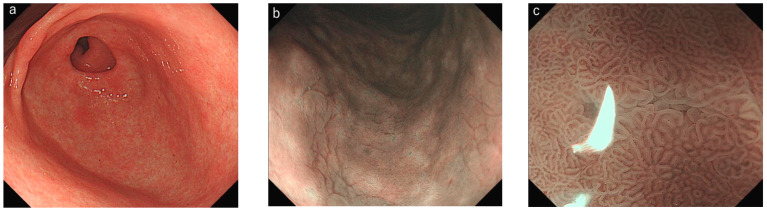
Case 1. (**a**) Antral part observed with white-light endoscopy. (**b**) Light-blue crest was observed in a focal area with nonmagnifying narrow-band imaging (NBI). (**c**) Only marginal turbid band was observed under magnifying NBI in this area.

**Figure 3:**
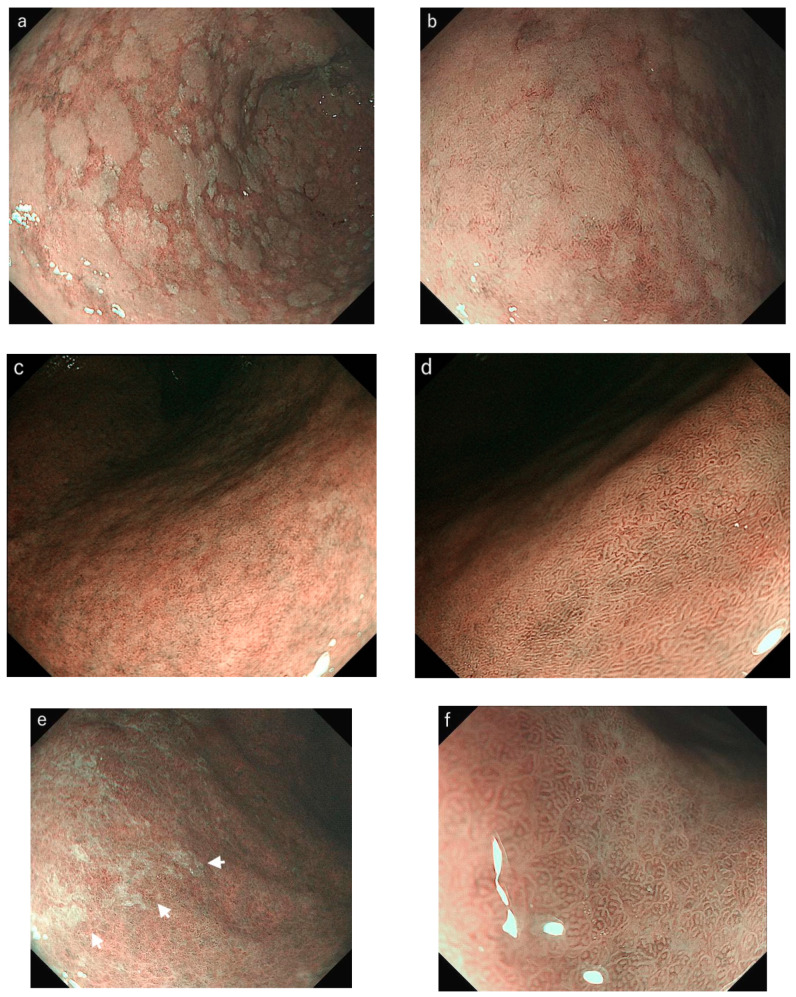
Case 2. (**a**,**b**) Antrum: white opaque substance was observed in an extensive area with nonmagnifying and magnifying narrow-band imaging (NBI). (**c**) Lesser curvature of the corpus: light-blue crest was observed in a focal area. (**d**) Tubulovillous microsurface structure was observed in an extensive area with magnifying NBI. (**e**,**f**) Greater curvature of the corpus: whitish mucus was misdiagnosed as the presence of white opaque substance with nonmagnifying NBI (white arrow). The endoscopic grading of gastric intestinal metaplasia score for nonmagnifying image-enhanced endoscopy (IEE) (total score of 6; antrum score of 2, 2; corpus score of 1, 1) was similar to that for magnifying IEE (total score of 6; antrum score of 2, 2; corpus score of 2, 0) in this case.

Of the total evaluated areas (*n* = 400), LBC scores of 0, 1, and 2 were found in 208 (52%), 147 (37%), and 45 (11%) areas in nonmagnifying IEE and in 223 (56%), 133 (33%), and 44 (11%) areas, respectively, in magnifying IEE (Table 2). WOS scores of 0, 1, and 2 were found in 317 (79%), 53 (13%), and 30 (8%) areas in nonmagnifying IEE and in 323 (81%), 47 (12%), and 30 (7%) areas in magnifying IEE, respectively (Table 3). In several cases, tubulovillous structure/MTB was misdiagnosed as the presence of LBC in nonmagnifying IEE (Figure 2). The whitish mucus observed on the gastric mucosa also presented a risk of being misdiagnosed as the presence of WOS in nonmagnifying IEE (Figure 3). The concordance rate between nonmagnifying and magnifying IEE was 80.5% and 93.3% for the LBC and WOS scores, respectively. The strength of agreement showed good reproducibility between nonmagnifying and magnifying IEE (weighted kappa values of 0.69 (good) and 0.83 (excellent) for the LBC and WOS score, respectively).

The numbers of patients with an individual EGGIM score of 0, 1–4, and 5–8 in nonmagnifying IEE were 18, 55, and 27, respectively, and those in magnifying IEE were 28, 51, and 21, respectively (Table 4). The concordance rate of EGGIM was 75%, and the weighted kappa value was 0.67 (good) for both nonmagnifying and magnifying IEE.

The prevalence of high EGGIM scores (5–8) in patients without and with GC was 22% and 45% in nonmagnifying IEE and 16% and 38% in magnifying IEE, respectively (Table 5). Patients with high EGGIM scores had a significant risk of GC in both nonmagnifying IEE (odds ratio (OR), 3.3; 95% confidence interval (CI), 1.2–9.0) and magnifying IEE (OR, 3.1; 95% CI, 1.1–8.9).

## 4. Discussion

To the best of our knowledge, this is the first study to investigate the clinical difference in IEE-IM between magnifying and nonmagnifying observations. However, previous studies regarding IEE-IM were conducted only with magnifying IEE. The results of our study indicated good reproducibility in the assessment of gastric IM between nonmagnifying and magnifying IEE. The findings of the present study may be useful in daily clinical practice, because they demonstrate that nonmagnifying IEE performed to assess gastric IM is beneficial for stratifying the risk of GC, similar to magnifying IEE (which cannot be used in all clinical settings).

Histopathology is the most popular method of assessing GC risk. The OLGIM classification, which is histologically validated via biopsy specimens for gastric IM according to the updated Sydney system, has shown a strong association with GC risk [2]. Among the OLGIM stages (I–IV), higher stages are significantly associated with an increased risk of GC and with higher ORs in stages III (OR = 5.5) and IV (OR = 8.91) [3]. MAPS II, the European endoscopic guideline for the diagnostic assessment of gastritis and stomach dysplasia, also strongly recommends histological confirmation of GC risk [17]. However, conventional endoscopic assessment for gastric IM is not recommended because of the poor correlation between histological and conventional WLE findings for gastric IM.

Several studies that focused on the diagnostic accuracy of NBI endoscopy for gastric IM showed an advantage of NBI over conventional WLE (94% in NBI, 11% higher than that in high-definition WLE) [6]. The EGGIM system with NBI has been proposed for staging gastritis and stratifying the risk of GC using IEE. This classification rates the entire gastric mucosa according to the extent of gastric IM and exhibits a good correlation with the histological OLGIM staging system [6]. Furthermore, high EGGIM scores were found to be a significant risk factor for GC in Western and Eastern countries [7,11]. Endoscopic assessment of gastric IM is superior, because it minimizes the risk of bleeding from multiple biopsies and requires less time and cost than does histopathology. Endoscopic assessments also enable the evaluation of the entire gastric mucosa, which may allow for a more accurate quantitative analysis of GC risk compared with point estimation, as in biopsies.

Magnifying IEE facilitates detailed observation of the gastric mucosa. In a previous multicenter study, magnifying IEE exhibited higher accuracy than conventional nonmagnifying WLE in diagnosing small GC [15]. Magnifying IEE can reveal the detailed microsurface structure and microvascular architecture of the gastric mucosa; it is therefore useful for diagnosing not only GC but also gastric precancerous conditions. Uedo et al. [8] reported that LBC is useful in diagnosing histological gastric IM with a sensitivity, specificity, and accuracy of 89%, 93%, and 91%, respectively. WOS was also found to be associated with histological gastric IM with 50% sensitivity and 100% specificity [10]. Moreover, the tubulovillous microsurface structure and MTB were reported to be associated with gastric IM [11,12,13].

These previous studies used magnifying IEE; however, magnifying endoscopy cannot be used for routine endoscopy in every clinic or general hospital. Thus, we evaluated the differences in the diagnosis of gastric IM between magnifying and nonmagnifying IEE. Our results suggest that nonmagnifying IEE, which can be used at most endoscopic facilities, is useful in assessing the risk of GC, similar to magnifying IEE. Moreover, nonmagnifying IEE has the advantage of assessing a wide range of view; by contrast, magnifying IEE has a limited field and is a lengthy procedure.

In this study, we investigated the concordance rate of the LBC and WOS scores in both magnifying and nonmagnifying IEE. Both LBC and WOS exhibited good reproducibility in magnifying and nonmagnifying IEE; however, the concordance rate and kappa value were higher in WOS (93.3% and 0.83, respectively) than in LBC (80.5% and 0.69, respectively). Several previous reports have addressed the interobserver concordance rate in IEE-IM. Kanemitsu et al. [10] demonstrated that the concordance rate between two endoscopists for the presence of WOS was 95.0% with a kappa value of 0.89 (excellent), whereas that for LBC was 72.5% with a kappa value of 0.43 (moderate). Pimentel-Nunes et al. [5] also reported a lower kappa value (0.49) in the diagnosis of LBC among experts, experienced observers, and inexperienced observers. In our study, WOS could be observed as clear-bordered, white areas even in nonmagnifying IEE and was easily recognized in most cases. Conversely, LBC was difficult to assess without magnification, because the blue line at the edge of the epithelial microsurface structure was observed as obscure blue areas. In several instances, tubulovillous structure/MTB was misdiagnosed as the presence of LBC in nonmagnifying IEE (Figure 2). This has been suggested to be the cause of the lower concordance rate in not only interobserver agreement but also between magnifying and nonmagnifying IEE findings.

Although each IEE-IM finding for diagnosing gastric IM demonstrated a characteristic difference between magnifying and nonmagnifying IEE, the stratification for the risk of GC using the EGGIM system was considered sufficient for both. The EGGIM system assesses the areas in which combinations of IEE-IM findings are present, including LBC, WOS, and tubulovillous/MTB. The accurate diagnosis of gastric IM with detailed micromucosal features using magnifying IEE is reliable; however, the assessment of the extent of uniformly distributed areas with white or blue color changes using nonmagnifying IEE is also considered sufficient to stratify the risk of GC.

Recent reports have indicated that artificial intelligence (AI) systems may be a powerful resource for endoscopic diagnosis of gastric precancerous lesions and *H. pylori* infection, with a pooled diagnostic accuracy of 90% and 80%, respectively [18]. In particular, the computer-aided detection system proposed in one study achieved diagnostic accuracy for gastric precancerous conditions comparable to that of experts and superior to that of nonexperts [19]. Furthermore, deep convolutional neural network recognition of atrophic gastritis and gastric IM was achieved in another study [20]. However, the European Society of Gastrointestinal Endoscopy guidelines recently addressed the potential usefulness of AI systems, stating that AI-assisted diagnosis of atrophy and IM should be comparable to that provided by the established biopsy protocol, including extent estimation and subsequent allocation to the appropriate endoscopic surveillance interval [21].

This study has several limitations. The main limitation is that a histological evaluation of gastric IM was not performed. Many reports have stated that magnifying IEE enables a highly accurate diagnosis of histological gastric IM using IEE-guided sampling of biopsy specimens. Our study suggests that nonmagnifying IEE has the potential to stratify the risk of GC and determine the extent of histological gastric IM, similar to magnifying IEE; however, further studies involving histological confirmation are warranted to validate this ability of nonmagnifying IEE. Second, this was a single-center study with a small number of recruited patients. Additionally, the small number of endoscopists who identified the differences in the magnifying and nonmagnifying IEE findings is considered to be a source of bias. Furthermore, we did not evaluate the interobserver agreement among the endoscopy experts, intermediates, and trainees because of the limited number of participants. Multicenter studies with more participants are warranted for the further validation of our findings. Third, we evaluated four areas of the stomach for the assessment of EGGIM in this study. The EGGIM score was originally proposed to involve the evaluation of five areas, including the incisura of the stomach, according to the updated Sydney system; however, we conducted our evaluation without the incisura because of the questionable diagnostic benefit of taking biopsy specimens from the incisura [14]. Although this simplified method has the potential to minimize the procedure time, further investigation is needed to clarify the difference in the risk stratification for GC between inclusion and exclusion of incisura assessment. Fourth, although IEE-IM was assessed using several magnified pictures that were believed to represent the IEE images in the field, the ability of magnifying IEE to assess only limited fields is considered a source of study bias, unlike nonmagnifying IEE. Thus, real-time and prospective cohort studies are needed to assess the differences in diagnosing gastric IM and GC risk between nonmagnifying and magnifying IEE.

## 5. Conclusions

High-resolution magnifying and nonmagnifying IEE demonstrated good agreement in the evaluation of endoscopic findings associated with gastric IM. Nonmagnifying IEE may have the potential to assess the extent of gastric IM and individual risk of GC, similar to magnifying IEE, which warrants further investigation with histological assessment.

## Figures and Tables

**Table 1:** Baseline clinical characteristics of the 100 patients included in this study.

Mean age (SD) (years)	63 (12.8)
Male (%)	57 (57)
Smoking (%)	46 (46)
Alcohol drinking (%)	62 (62)
Endoscopic indication (%) Symptomatic Screening/surveillance/pretreatment evaluation	21 (21) 79 (79)
Acid suppressant intake (%)	16 (16)
History of *Helicobacter pylori* eradication therapy (%)	11 (11)
*H. pylori* status No infection Current infections Past infection	21 (21) 59 (59) 20 (20)
Endoscopic atrophy (Kimura–Takemoto classification) None-Mild (C0, C1) Moderate (C2, C3) Severe (O1, O2, or O3)	22 (22) 46 (46) 32 (32)
Patients with gastric cancer	21 (21)

SD, standard deviation.

**Table 2:** LBC in all 400 observed areas under nonmagnifying and magnifying IEE.

		LBC in Magnifying IEE		
		None (*n* = 223)	Focal (*n* = 133)	Extensive (*n* = 44)
LBC in nonmagnifying IEE (%)	None (*n* = 208)	193 (93)	13 (6)	2 (1)
	Focal (*n* = 147)	24 (16)	105 (72)	18 (12)
	Extensive (*n* = 45)	6 (13)	15 (33)	24 (54)

LBC, light-blue crest; IEE, image-enhanced endoscopy.

**Table 3:** WOS in all 400 observed areas under nonmagnifying and magnifying IEE.

		WOS in Magnifying IEE		
		None (*n* = 323)	Focal (*n* = 47)	Extensive (*n* = 30)
WOS in nonmagnifying IEE (%)	None (*n* = 317)	310 (98)	6 (1.8)	1 (0.2)
	Focal (*n* = 53)	11 (21)	38 (72)	4 (7)
	Extensive (*n* = 30)	2 (7)	3 (10)	25 (83)

WOS, white opaque substance; IEE, image-enhanced endoscopy.

**Table 4:** Individual EGGIM score in nonmagnifying and magnifying IEE.

		EGGIM Score in Magnifying IEE		
		0 (*n* = 28)	1–4 (*n* = 51)	5–8 (*n* = 21)
EGGIM score in nonmagnifying IEE (%)	0 (*n* = 18)	18 (100)	0 (0)	0 (0)
	1–4 (*n* = 55)	9 (16)	41 (74)	5 (9)
	5–8 (*n* = 27)	1 (4)	10 (37)	16 (59)

EGGIM, endoscopic grading of gastric intestinal metaplasia; IEE, image-enhanced endoscopy.

**Table 5:** EGGIM score in patients with and without GC in nonmagnifying and magnifying IEE.

		Number (%)		Odds Ratio (95% CI)	*p* Value
		Patients with Non-GC	Patients with GC		
EGGIM score in nonmagnifying IEE (%)	Low (0–4)	62 (78)	11 (55)	1	
	High (5–8)	17 (22)	10 (45)	3.3 (1.2–9.0)	0.017
EGGIM score in magnifying IEE (%)	Low (0–4)	66 (84)	13 (62)	1	
	High (5–8)	13 (16)	8 (38)	3.1(1.1–8.9)	0.030

EGGIM, endoscopic grading of gastric intestinal metaplasia; GC, gastric cancer; IEE, image-enhanced endoscopy; CI, confidence interval.

## Data Availability

Not applicable.
